# Regulation by anti-inflammatory cytokines (IL-4, IL-10, IL-13, TGFβ)of interleukin-8 production by LPS- and/ or TNFα-activated human polymorphonuclear cells

**DOI:** 10.1155/S0962935196000488

**Published:** 1996-10

**Authors:** C. Marie, C. Pitton, C. Fitting, J-M. Cavaillon

**Affiliations:** 1 Unité d'lmmuno-Allergie Institut Pasteur 28 rue Dr Roux Paris cedex 15 75724 France

## Abstract

The capacity to down-regulate the production of IL-8 by LPS-activated human polymorphonuclear cells (PMN) has been demonstrated for IL-4, IL-10, and TGFβ. We compared their relative capacities and further extended this property to IL-13. We report a great heterogeneity among individuals related to the responsiveness of PMN to the IL-4 and IL-13 inhibitory effects while their response to the IL-10 effect was homogenous. The inhibitory activities were observed at the transcriptional level. IL-8 induction by TNFα was, unlike its induction by LPS, resistant to the inhibitory effects of IL-10, IL-4, IL-13 and TGFβ. Furthermore, IL-10 and IL-4 inhibitory activity were less effective when TNFα was acting synergistically with LPS to induce IL-8 production by PMN. LPS-induced cell-associated IL-8, detected in the PMN cultures, could be marginally inhibited by IL-4 and IL-10. Altogether, our data demonstrate that IL-13 is able to inhibit LPS-induced IL-8 production by human PMN, although IL-10 remains the most active anti-inflammatory cytokine. Despite the capacity of IL-4, IL-10, and IL-13 to limit the production of TNFα-induced IL-8 in a whole blood assay, none was able to inhibit this production when studying isolated human polymorphonuclear cells.


Mediators of Inflammation, 5, 334–340 (1996)

### Regulation by anti-inflammatory cytokines (IL-4, IL-10, IL-13, TGFβ) of interleukin-8 production by LPS- and/or TNFα-activated human polymorphonuclear cells

C. Marie, C. Pitton, C. Fitting and J-M. Cavaillon[CA]

Unité d'Immuno-Allergie, Institut Pasteur, 28 rue Dr Roux, 75724 Paris cedex 15, France

[CA]Corresponding Author
Tel: (+33) 01 45 68 82 38
Fax: (+33) 01 40 61 31 60
Email: jmcavail@pasteur.fr

**The capacity to down-regulate the production of IL-8 by LPS-activated human polymorphonuclear cells (PMN) has been demonstrated for IL-4, IL-10, and TGFβ. We compared their relative capacities and further extended this property to IL-13. We report a great heterogeneity among individuals related to the responsiveness of PMN to the IL-4 and IL-13 inhibitory effects while their response to the IL-10 effect was homogenous. The inhibitory activities were observed at the transcriptional level. IL-8 induction by TNFα was, unlike its induction by LPS, resistant to the inhibitory effects of IL-10, IL-4, IL-13 and TGFβ. Furthermore, IL-10 and IL-4 inhibitory activity were less effective when TNFα was acting synergistically with LPS to induce IL-8 production by PMN. LPS-induced cell-associated IL-8, detected in the PMN cultures, could be marginally inhibited by IL-4 and IL-10. Altogether, our data demonstrate that IL-13 is able to inhibit LPS-induced IL-8 production by human PMN, although IL-10 remains the most active anti-inflammatory cytokine. Despite the capacity of IL-4, IL-10, and IL-13 to limit the production of TNFα-induced IL-8 in a whole blood assay, none was able to inhibit this production when studying isolated human polymorphonuclear cells.**

**Key words:** Cell-associated cytokines, Cytokine network, Endotoxins, Inflammation, Neutrophils

### Introduction

Inflammation is characterized by a complex interaction of pro- and anti-inflammatory mediators. A favourable balance leads to a recovery of homeostasis while an unfavourable balance, associated with an exacerbated release of pro-inflammatory cytokines, leads to deleterious effects. Individual background seems to govern this equilibrium. In man, for example, it is known that a great heterogeneity exists between individuals in terms of their levels of production of TNFα by LPS-stimulated monocytes.[1] Similarly, the capacity to produce the Th2 cytokines, including the anti-inflammatory IL-4 and IL-10 cytokines, rather than the Th1-derived cytokines which include the pro-inflammatory interferon-γ, seems to be genetically determined.[2] Furthermore, the sensitivity of target cells to cytokine signals constitutes another level of heterogeneity.[3] The capacity of IL-4, IL-10, IL-13 and TGFβ to inhibit the synthesis of pro-inflammatory cytokines by activated monocytes/macrophages has been well established.[4–7] However, the mononuclear phagocytes are not the unique source of IL-1, TNFα, IL-6 and IL-8, and many other cell types can contribute to the inflammatory process as a consequence of their ability to produce pro-inflammatory cytokines. Upon activation, polymorphonuclear cells (PMN) are a major source of mediators, including eicosanoids, proteolytic enzymes and free radicals, all of which contribute to the exacerbation of inflammatory processes. *In vivo* models of infection have clearly established that PMNs do contribute to tissue injury and organ dysfunction and are involved in the development of fatal complication in sepsis syndrome.[8–10] Their capacity to be activated by cytokines following the release of bacterial derived substances has been illustrated in *in vivo* models of neutrophil depletion.[11,12] Indeed, many substances, including opsonized yeast, bacterial-derived products—such as endotoxin (LPS, lipopolysaccharide)—and cytokines—such as IL-1, IL-2 and TNFα—are able to trigger PMN, leading to a notable production of IL-8.[13–16] IL-8, being a potent chemokine and activator of PMN, has emerged in recent years as a pivotal factor of inflammation. The presence of IL-8 *in vivo* has been clearly associated with neutrophil recruitment[17–19] and the sever-

© 1996 Rapid Science Publishers ity of the pathology.[20–28] Indeed, levels of IL-8 in broncho-alveolar lavage fluids and in plasma of septic patients correlate with the clinical outcome.[23–28] In addition, experiments using anti-IL-8 antibodies have clearly demonstrated the contribution of IL-8 in the inflammatory process.[29–31] In the present study we have compared the relative capacities of IL-4, IL-10, IL-13 and TGFβ to interfere with IL-8 production by activated human PMN. This is the first report on the individual heterogeneity related to the inhibitory activities of IL-4 and IL-13. In addition, we demonstrated that these cytokines are devoid of any activity when TNFα was employed as the triggering agent, and showed that the inhibitory activity of IL-10 on LPS-induced IL-8 was significantly hampered by the presence of TNFα.

### Material and Methods

### Human polymorphonuclear cells

Blood was drawn from human volunteers (Fondation Nationale de Transfusion Sanguine, Paris) on heparin (20 IU/ml). Ten volumes of blood were mixed with 2 volumes of glucose dextran (3% glucose; 3% dextran T250 Pharmacia, Sweden), and the leukocytes recovered following a 40 min sedimentation at room temperature. Leukocytes were then diluted 1:2 in RPMI-1640 medium and layered on Ficoll-Hypaque (Milieu de Séparation des Lymphocytes, MSL, Eurobio, Les Ulis, France). The ratio was 2 volumes of leukocytes to 1 volume of MSL. After centrifugation for 25 min at 15°C and 500 × *g*, the pellet was washed and centrifuged once 5 min at 300 × *g*. Contaminating erythrocytes were lysed following a 5 min incubation at 4°C of the cell pellet resuspended in 5 ml of lysis buffer ($NH_4Cl$ = 8.32 g/l; $NaHCO_3$ = 0.84 g/l; $Na_4EDTA$ = 43.2 mg/l). Lysis was stopped by adding a large excess of RPMI and the cells washed and centrifuged for 10 min at 200 × *g*. The viability of polymorphonuclear cells (PMN) was assessed by numerating the cells in 0.1% eosine. A non-specific esterase staining was performed to evaluate the monocyte contamination which never exceeded 0.5%.

### In vitro culture

For whole blood assays, blood was diluted 1:5 in RPMI-1640 medium (Bio-Whittaker, USA) supplemented with L-glutamine (300 mg/l) and antibiotics (penicillin, 100 IU/ml; streptomycin, 100 μg/ml), and 0.5 ml cultured per well in 24-well multidish plates (Nunclon, Rockville, Denmark), at 37°C in a 5% $CO_2$ incubator for 18–24 h.

PMN ($2 \times 10^6$ cells/ml) were cultured in RPMI-1640 medium supplemented with L-glutamine, antibiotics and 5% heat inactivated normal human serum (a pool of sera from healthy volunteers). 0.5 ml per well in 24-well multidish plates of PMN suspension were incubated for 18–24 h at 37°C in a 5% $CO_2$ incubator. The cell viability after 24 h ranged from 64% to 84%. Stimuli were added at the beginning of the culture in a volume not exceeding 10 μl. At the end of the culture, the supernatants were harvested, centrifuged 10 min at 300 × *g* and 15°C and kept at −20°C before cytokine assessments. In some experiments, at the end of the culture period, the cells were lysed by adding 100 μl of lysis buffer to the cell pellet (TRAx® buffer, T-Cell Diagnostic, USA) before adding 100 μl of diluent buffer (TRAx® buffer, T-Cell Diagnostic, USA) and 300 μl of RPMI medium.

### Reagents

*Escherichia coli* (0111:B4) lipopolysaccharide was purchased from Sigma (USA). Recombinant human IL-10 ($1-2 \times 10^7$ U/mg) and IL-13 were a generous gift of Dr John Abrams (DNAX, Palo Alto, USA) and Dr Adrian Minty (Sanofi Recherche, Labège, France), respectively. Similar results have been obtained with IL-10 and IL-13 from R&D Systems (USA). TNFα was obtained from Rhône Poulenc (France) and IL-4 and TGFβ ($2 \times 10^7$ U/mg) were purchased from Immugenex (USA) and R&D systems (USA), respectively.

### IL-8 ELISA

IL-8 ELISA was performed as previously described[28] using a monoclonal anti-human IL-8 antibody and a rabbit polyclonal anti-IL-8 antibody kindly provided by N. Vita (Sanofi Recherche, Labège, France).

### Northern blot analysis

Total cellular RNA from $20-30 \times 10^6$ PMN was extracted using the guanidine isothiocyanate method.[32] 5–10 μg total RNA per wells were separated on 1% agarose gels containing 6.6% formaldehyde, transferred to Hybond-$N^+$ membranes (Amersham) and hybridized with $^{32}P$-labelled IL-8 and β-actin probes. The human IL-8 cDNA probe, generously provided by Dr J. Oppenheim (NCI, Frederick, MD), was a purified 0.5 kb *Eco*R I fragment. The mouse β-actin cDNA probe was a 0.97 kb *PST* I fragment.

Membranes were washed and exposed to X-ray films (Kodak XAR-5) for 24–48 h at −70°C in the presence of an intensifying screen. Relative signal strength was measured by laser densitometry (Ultroscan XL, Pharmacia). The ratio IL-8: β-actin mRNA was then calculated.

### Statistical analysis

Results are expressed as mean ± SEM. Statistical analysis was performed using the Wilcoxon signed-rank test.

### Results

### Inhibition of IL-8 production by IL-13 compared with other anti-inflammatory cytokines

As shown in Fig. 1A, IL-4, IL-10, and IL-13 are able to inhibit in a dose-dependent fashion the IL-8 production in a whole blood assay triggered by LPS. The results are the mean of experiments performed with five different donors and were homogeneous. This was not the case when these cytokines were studied with isolated human polymorphonuclear cells (see below, the individual responsiveness). A dose-response curve of the inhibitory effect of IL-4, IL-10 and IL-13 on LPS-induced IL-8 production by activated human PMN from one given donor is shown in Fig. 1B. IL-10, IL-4 and IL-13 were able, in a dose-dependent fashion, to inhibit the release of IL-8 by LPS-activated PMN. In the whole blood assay as well as with the isolated PMN, no further inhibitory effects could be obtained with 30 ng/ml of cytokines, compared with 10 ng/ml, and subsequently, a fixed cytokine concentration of 10 ng/ml was used.

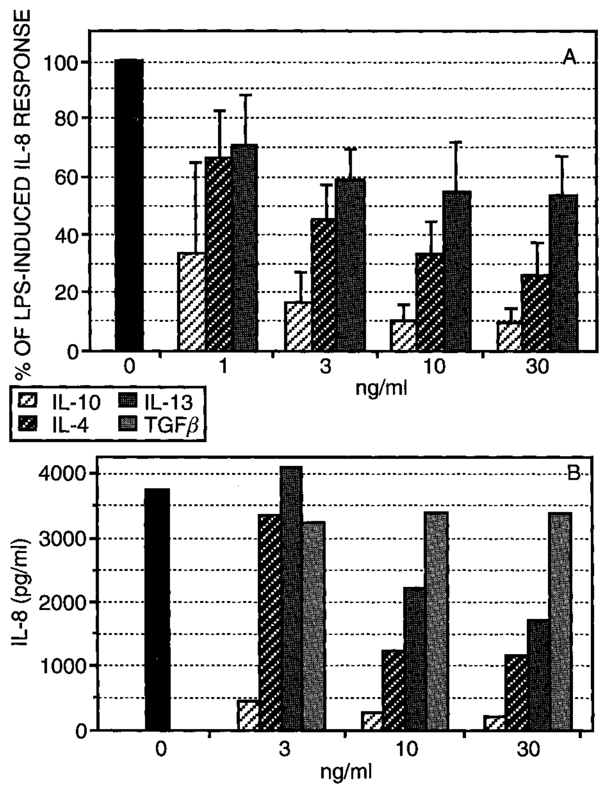

FIG. 1. (A) Dose-response curve of the inhibitory effects of IL-4, IL-10 and IL-13 on IL-8 production in a whole blood assay. Cells were stimulated by 100 ng/ml *Escherichia coli* LPS. The data are the mean ± SEM of five different experiments. (B) Dose-response curve of the inhibitory effects of IL-4, IL-10, IL-13 and TGFβ on IL-8 production by LPS activated PMN. Cells from one donor for whom IL-4 and IL-13 had inhibitory activity were activated by 100 ng/ml *E. coli* LPS.

Neither IL-10 nor IL-13 had any effect on the 'spontaneous' IL-8 production by human PMN (262 ± 64 pg/ml and 240 ± 41 pg/ml, respectively *vs* 191 ± 39 pg/ml; $n = 12$). IL-4 had a weak but significant capacity to limit the spontaneous production of IL-8 (103 ± 20 pg/ml; $p < 0.01$) while TGFβ amplify it (588 ± 234 pg/ml; $p = 0.05$).

Inhibitory activities of IL-10, IL-4, IL-13 and TGFβ were compared on PMN activated by either LPS or TNFα. As shown in Fig. 2A, the studies on the inhibition of LPS-induced IL-8 production performed with the cells from different donors, revealed that IL-10 at 10 ng/ml was very efficient whereas similar amounts of IL-4 were less active (81% inhibition *vs* 37% inhibition; $p < 0.01$). IL-13 and TGFβ, with respectively 16% and 12% inhibition, were very weakly inhibitory, although this was statistically significant in view of the large number of experiments performed ($n = 12$).

When TNFα was employed as the stimulating agent, IL-10 had a high inhibitory activity, and IL-4 and IL-13 had similar capacity to counteract the production of IL-8 by whole blood cells, while TGFβ was devoid of any inhibitory activity (data not shown). In contrast to the results obtained in whole blood assays, none of the anti-inflammatory cytokines were able to counteract the IL-8 production induced by TNFα (Fig. 2B).

### Synergy between LPS and TNF: effect of anti-inflammatory cytokines

Since cells are most likely exposed to many triggering signals during infection by Gram negative bacteria, it was interesting to investigate the capacity of the anti-inflammatory cytokines to reduce the IL-8 production stimulated by both LPS and TNFα. The results are shown in Fig. 2C. The activation of whole blood cells by both LPS and TNFα led to additive IL-8 production (data not shown), while a synergy

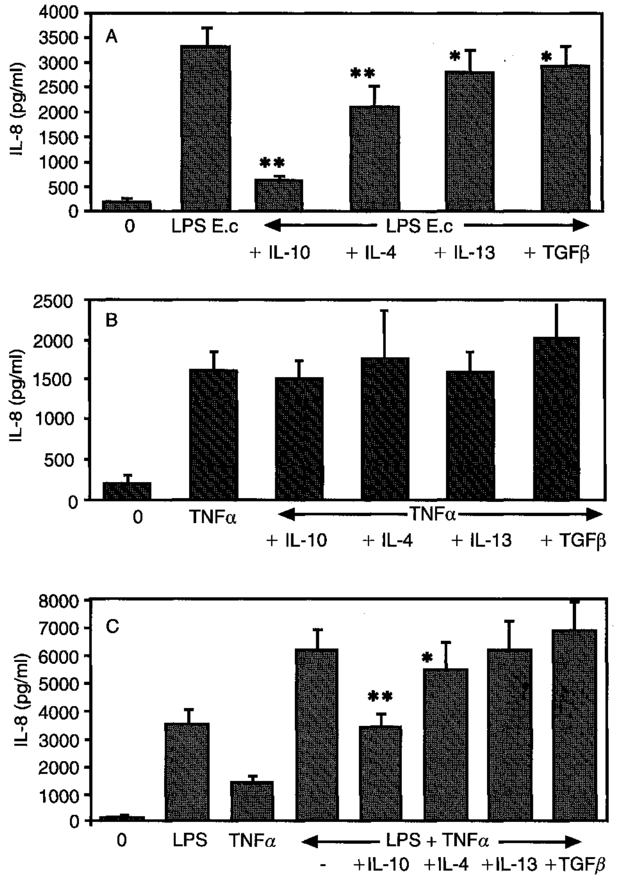

FIG. 2. Comparative inhibitory activities of the same concentration (10 ng/ml) of IL-10, IL-4, IL-13 and TGFβ on LPS-induced (100 ng/ml) (A), TNFα-induced (10 ng/ml) (B), and on the combination of 100 ng/ml *Escherichia coli* LPS and 10 ng/ml TNFα-induced (C) IL-8 production by human PMN. The results are expressed as the mean ± SEM of 12 (A), 8 (B), and 10 (C) different experiments (* $p < 0.05$; ** $p < 0.01$ *vs* LPS alone, TNF alone or LPS + TNF).

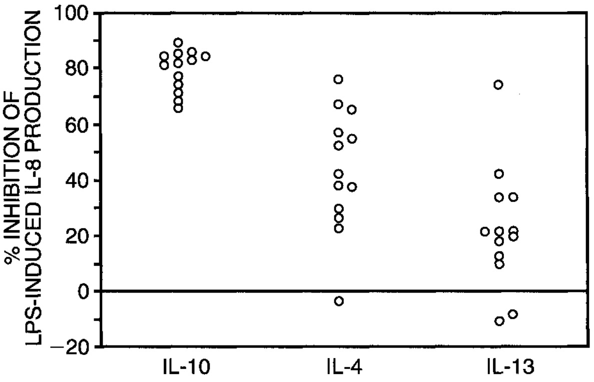

FIG. 3. Comparative inhibitory activities for individual donors of the same concentration (10 ng/ml) of IL-10, IL-4, IL-13 on IL-8 production by human PMN stimulated by 100 ng/ml *Escherichia coli* LPS. Results are expressed as percentage of inhibition and each symbol represents a separate donor.

was noticed when PMN were studied (arithmetic sum = 5007 ± 514 pg/ml *vs* experimental data = 6225 ± 651 pg/ml, $n = 17$, $p < 0.02$). In whole blood cells, IL-10 and IL-4 were particularly efficient in inhibiting IL-8 production (data not shown). On the contrary with PMN, only IL-10 dramatically inhibited IL-8 production, while IL-4 was weakly inhibitory, and IL-13 and TGFβ were not significantly inhibitory. However, the inhibitory activity of IL-10, which was higher than 80% when LPS was used alone, dropped to only 45% (for the same concentration of 10 ng/ml) when both activators were employed.

### Individual responsiveness

When the results were individually plotted (Fig. 3), it was noticed that the inhibitory effect of IL-10 was very similar between donors (range: from 65% to 90% inhibition) whilst the effect of IL-4 varied greatly depending on donor (range: from 0% to 78% inhibition). Similarly, IL-13-induced inhibition varied considerably between donors. A weak, but significant correlation exists between the potency of IL-4 and IL-13 to inhibit the IL-8 production by LPS-stimulated PMN ($r = 0.52$, $p < 0.05$).

### Detection of cell-associated IL-8

When cell-associated IL-8 was assessed, high concentrations of this cytokine could be detected in the PMN lysates obtained with the TRAx® buffer, independently of the triggering signal (Table 1). To further evaluate the proportion of the cytokine linked to its receptor on PMN, IL-8 was measured following treatment of cells with a glycine-HCl buffer. One-fifth of the cell-associated IL-8 was recovered with such a treatment (data not shown).

IL-4, IL-10 and IL-13 but not TGFβ could also reduce the presence of cell-associated IL-8 in the PMN cultures (Table 2). However, the reduction was less pronounced than when studying released IL-8 and did not reach statistical significance.

### Inhibition of IL-8 mRNA accumulation induced by LPS

To determine whether the anti-inflammatory cytokines act at the transcriptional level, Northern blot analysis of IL-8 mRNA were performed

**Table 1.** Released and cell-associated IL-8 (pg/ml) induced by LPS and TNFα in neutrophil cultures (n = 5)

| Inducers | Released IL-8 | Cell-associated IL-8 |
|---|---|---|
| None | 275 ± 61 | 1 635 ± 716 |
| LPS *Escherichia coli* (100 ng/ml) | 6 010 ± 1 261 | 8 455 ± 2 163 |
| TNFα (10 ng/ml) | 1 417 ± 431 | 3 967 ± 213 |

**Table 2.** Effects of IL-4, IL-10, IL-13 and TGFβ on released and cell-associated IL-8 (pg/ml) production induced by LPS in neutrophil cultures

| Addition to the culture | Released IL-8 | Cell-associated IL-8 |
|---|---|---|
| None | 335 ± 46[a] | 1 266 ± 266 |
| LPS *Escherichia coli* (100 ng/ml) | 8 120 ± 1 839 | 9 340 ± 2 762 |
| LPS + IL-4 (10 ng/ml) | 3 394 ± 787 | 5 455 ± 1 592 |
| LPS + IL-10 (10 ng/ml) | 2 732 ± 1 223 | 4 870 ± 1 078 |
| LPS + IL-13 (10 ng/ml) | 4 408 ± 652 | 7 499 ± 2 850 |
| LPS + TGFβ (10 ng/ml) | 6 026 ± 1 227 | 9 344 ± 3 661 |

[a]Mean ± SEM of five different experiments.

on total RNA isolated from PMN incubated for 5 h in the presence of LPS alone or LPS plus anti-inflammatory cytokines. No further significant effects could be observed at earlier time (data not shown). As shown in Fig. 4A, LPS alone or LPS + TGFβ led to an accumulation of IL-8 mRNA. No such accumulation was observed upon the addition of IL-10, IL-4 and IL-13. When analysing the experiments performed with five different donors, a statistically significant effect of IL-10 and IL-4 was noticed ($p = 0.04$), while a trend was observed with IL-13 ($p = 0.08$). The effect of IL-13 was observed in four out of five different experiments in agreement with the individual heterogeneity as assessed at the protein level.

### Discussion

The intensity of the inflammatory response is the result of a complex balance between pro- and anti-inflammatory mediators. Among the set of cytokines, IL-1, IFNγ, and TNFα orchestrate the inflammatory process, whilst IL-4, IL-10, IL-13, and TGFβ have been characterized as anti-inflammatory agents. This latter group is able to reduce the production of the pro-inflammatory cytokines by monocytes/macrophages[4–7] and also to interfere with some of their activities. In the present study we have compared the capacity of these anti-inflammatory cytokines to inhibit the production of IL-8 by isolated polymorphonuclear cells stimulated by LPS and/or TNFα. Human polymorphonuclear cells, the main target cells for IL-8, can be activated to release IL-8 following stimulation by LPS and/or TNFα. These results suggest that, following their recruitment in inflammatory tissues and local activation by bacterial-derived products or cytokines, PMN can perpetuate the chemoattractant process and cellular activation.

We observed that the production of IL-8 by LPS-activated human polymorphonuclear cells could be down-regulated by IL-10, IL-4, IL-13 and to a lesser extent by TGFβ. This is in

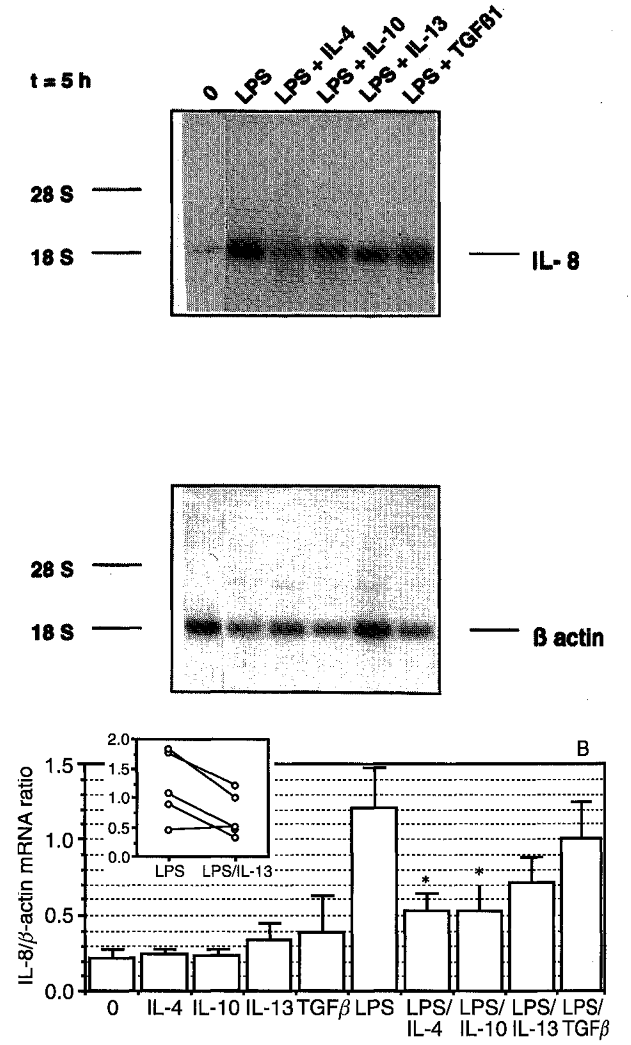

FIG. 4. (A) Northern blot analysis of IL-8 and β-actin mRNA expression in LPS- (100 ng/ml) activated human PMN in the absence or presence of anti-inflammatory cytokines (10 ng/ml). Both IL-8 and β-actin mRNA were quantified by laser densitometry; the IL-8; β-actin ratios were 0.05 for control (0), 0.89 for LPS, 0.25 for LPS + IL-4, 0.34 for LPS + IL-10, 0.33 for LPS + IL-13, and 0.89 for LPS + TGFβ. (B) Means of IL-8: β-actin ratios of five experiments performed with PMN from different donors (inset: individual results for the experiments performed with LPS and LPS + IL-13) (* $p < 0.05$ *vs* LPS).

agreement with the results of Kasama *et al.*[33] and Cassatella *et al.*,[34] who reported the capacity of IL-10 to limit IL-8 production, and with those of Wertheim *et al.*[35] who obtained similar results with IL-4. We observed that IL-4 was less efficient than IL-10 in agreement with the report of Wang *et al.*[36] In addition, when comparing the efficiency of the various anti-inflammatory cytokines, we have clearly shown a profound variability of PMN sensitivity to IL-4 and IL-13 between individuals. In contrast, IL-10 was uniformly active. These results probably do not reflect differences at the IL-4 receptor levels since there was no correlation between the levels of IL-8 and IL-1ra released in response to

LPS plus IL-4.[37] In preliminary experiments, it seemed that the individual responsiveness to IL-4 might vary with time since the percentage of inhibition was not reproducible with the cells prepared from the same donor at different time intervals. Although IL-13 shares many activities with IL-4, in most experiments IL-13 appeared less active than IL-4. The observed inhibitory activity was also observed at the transcriptional level. In a recent report, Girard *et al.*[38] showed that IL-13 itself could induce IL-8 production by human PMN. We never observed such induction at the protein level ($n = 12$ different donors). We are unable to explain such discrepancy, the only differences noted being the origin of the recombinant cytokines and the culture conditions. Further experiments should be performed to address this issue.

The up- and down-regulation of TNFα-induced IL-8 production by PMN has been rarely studied.[34] Interestingly, none of the tested anti-inflammatory cytokines were able to inhibit the production of IL-8 by PMN when TNFα was used as the stimulus. This observation seems to be linked to an intrinsic property of PMN since IL-10, IL-4 and IL-13 were able to significantly inhibit the IL-8 production by whole blood cells activated by TNFα (data not shown). However, IL-4 and IL-10 can modulate some PMN functions when activated by TNFα. Indeed, we have previously shown that IL-1ra production by TNFα-activated PMN could be up-regulated by these cytokines.[37] As shown in Table 3, the down-regulation of IL-8 production by PMN by the so-called anti-inflammatory cytokines, as well as the up-regulation of IL-1ra production is dependent on the nature of the triggering signal and these modulations are not correlated. Thus, the modulation of the cytokine production by IL-4, IL-10 or IL-13 is dependent on the nature of the stimuli. In addition, the nature of the activated cells play a crucial role as illustrated by the fact that IL-4 and IL-10 which are well known to inhibit the LPS-induced IL-8 production by monocytes–macrophages,[4–7] enhance it when acting on endothelial cells.[39]

**Table 3.** Effect of IL-4, IL-10, IL-13 and TGFβ on the production of IL-8 and IL-1ra by polymorphonuclear cells activated by either LPS or TNFα

| PMN activators | Produced cytokines | Anti-inflammatory cytokines | | | |
|---|---|---|---|---|---|
| | | IL-4 | IL-10 | IL-13 | TGFβ |
| LPS | IL-8[a] | ↓↓[c] | ↓↓↓ | ↓ | ↓ |
| | IL-1ra[b] | ↑↑ | 0 | 0 | 0 |
| TNF | IL-8[a] | 0 | 0 | 0 | 0 |
| | IL-1ra[b] | ↑↑ | ↑↑ | 0 | 0 |

[a]The present study.[37]
[b]Marie *et al.* (1996).
[c]↓, ↑ or 0 indicate reduction, enhancement, or absence of significant effect on cytokine production. The number of arrows is related to the intensity of the phenomenon.

We have also investigated the simultaneous action of LPS and TNFα on IL-8 production, particularly since PMN in infectious sites may well be exposed to both agents. In these experimental conditions, only IL-10 retained its ability to inhibit the IL-8 production by activated PMN, whereas IL-4 was far less active and IL-13 had no more inhibitory activity. However the inhibitory effect of IL-10 was far less pronounced than when LPS was used alone as a stimulus (48–58% *vs* 83% inhibition).

A significant amount of cell-associated IL-8 was also observed in the PMN culture, which regularly exceeded that found in the supernatants. The presence of IL-8 in lysates of activated PMN might be explained by the presence of receptor-bound IL-8 and by the presence of IL-8 in the intracellular compartments, resulting from secretory mechanisms and/or following the rapid internalization of the cytokine bound to its receptors.[40] As shown by Kuhns and Gallin, the accumulation of IL-8 within PMN is localized to a subcellular fraction of heterogenous light membranous organelles.[41] In addition, the authors suggested that IL-8 accumulation was under translational control. The fact that the anti-inflammatory cytokines had a minor effect on the cell-associated form of IL-8 suggests that most of its detection corresponds to the rapid internalization of surrounding IL-8 which is still present in the culture medium, independently of the presence of IL-4, IL-10 or IL-13.

In conclusion this is the first report on the ability of IL-13 to down-modulate the production of IL-8 by LPS-activated PMN in comparison with the other anti-inflammatory cytokines. In addition, we showed an individual responsiveness to the inhibitory activity of IL-4 and IL-13 on IL-8 production by LPS-activated PMN, whereas IL-10 was uniformly active. It appeared that IL-10 was the most active cytokine when LPS was employed as the stimulating agent, while its inhibitory property could not be observed when TNFα was the activator and was minimized when TNFα was used together with LPS. This late observation may well be relevant to some *in vivo* situations where recruited PMN encountered *in situ* various stimulating signals.

### References

1. Cavaillon JM, Tamion F, Marty C, Misset B, Fitting C, Carlet J. Multiorgan dysfunction and the implication of cytokines. In: Mutz NJ, Koller W,

Benzer H, eds. *Proceedings 7th European Congress on Intensive Care Medicine*. Bologna: Monduzzi Editore, 1994; 23–32.

2. Romagnani S. Induction of Th1 and Th2 responses: a key role for the natural immune response? *Immunol. Today* 1992; **13**: 379–381.
3. Bender JR, Sadeghi MM, Watson C, Pfau S, Pardi R. Heterogeneous activation thresholds to cytokines in genetically distinct endothelial cells: evidence for diverse transcription responses. *Proc Natl Acad Sci USA* 1994; **91**: 3944–3998.
4. Weiss L, Haeffner-Cavaillon N, Cavaillon JM, Kazatchkine MD. Human T cells and interleukin-4 inhibit the release of interleukin-1 induced by lipopolysaccharide in serum-free cultures of autologous monocytes. *Eur J Immunol* 1989; **19**: 1347–1350.
5. de Waal Malefyt R, Abrams J, Bennett B, Figdor CG, de Vries JE. Interleukin-10 inhibits cytokine synthesis by human monocytes: an autoregulatory role of IL-10 produced by monocytes. *J Exp Med* 1991; **174**: 1209–1220.
6. de Waal Malefyt R, Figdor CG, Huijbens R, Mohan-Peterson S, Bennett B, Culpepper J, Dang W, Zurawski G, de Vries JE. Effects of IL-13 on phenotype, cytokine production, and cytotoxic function of human monocytes. Comparisons with IL-4 and modulation by IFNγ or IL-10. *J Immunol* 1993; **151**: 6370–6381.
7. Bogdan C, Paik J, Vodovotz Y, Nathan C. Contrasting mechanisms for suppression of macrophage cytokine release by transforming growth factor-β and interleukin-10. *J Biol Chem* 1992; **267**: 23301–23308.
8. Sato T, Shinzawa H, Abe Y, Takahashi T, Arai S, Sendo F. Inhibition of *Corynebacterium parvum*-primed and lipopolysaccharide-induced hepatic necrosis in rats by selective depletion of neutrophils using a monoclonal antibody. *J Leuk Biol* 1993; **53**: 144–150.
9. Hewett JA, Schultze AE, VanCise S, Roth RA. Neutrophil depletion protects against liver injury from bacterial endotoxin. *Lab Invest* 1992; **66**: 347–361.
10. Nuijens JH, Abbink JJ, Wachtfogel YT, Colman RW, Eerenberg AJM, Dors D, Kamp AJM, Strack van Schijndel RJM, Hack CE. Plasma elastase α1-antitrypsine and lactoferrin in sepsis: evidence for neutrophils as mediators in fatal sepsis. *J Lab Clin Med* 1992; **119**: 159–168.
11. Mallick AA, Ishizaka A, Stephens KE, Hatherill JR, Tazeloor HD, Raffin TA. Multiple organ damage caused by tumor necrosis factor and prevented by prior neutrophil depletion. *Chest* 1989; **95**: 1114–1120.
12. Horgan MJ, Palace GP, Everitt JE, Malik AB. TNF release in endotoxemia contributes to neutrophil-dependent pulmonary edema. *Am J Physiol* 1993; **264**: H1161–H1165.
13. Bazzoni F, Cassatella MA, Rossi F, Ceska M, Dewald B, Baggiolini M. Phagocytosing neutrophils produce and release high amounts of interleukin-8. *J Exp Med* 1991; **173**: 771–774,.
14. Strieter RM, Kasahara K, Allen RM, Standiford TJ, Rolfe MW, Becker FS, Chensue SW, Kunkel SL. Cytokine-induced neutrophil-derived interleukin-8. *Am J Pathol* 1992; **141**: 397–407.
15. Fujishima S, Hoffman AR, Vu T, Kim KJ, Zheng H, Daniel D, Kim Y, Wallace EF, Larrick JW, Raffin TA. Regulation of neutrophil interleukin-8 gene expression and protein secretion by LPS, TNFα and IL-1β. *J Cell Physiol* 1993; **154**: 478–485.
16. Wei S, Liu JH, Blanchard DK, Djeu JY. Induction of IL-8 gene expression in human polymorphonuclear neutrophils by recombinant IL-2. *J Immunol* 1994; **152**: 3630–3636.
17. Foster SJ, Aked DM, Schröder JM, Christophers E. Acute inflammatory effects of a monocyte-derived neutrophil-activating peptide in rabbit skin. *Immunology* 1989; **67**: 181–183.
18. Colditz I, Zwahlen R, Dewald B, Baggiolini M. *In vivo* inflammatory activity of neutrophil-activating factor, a novel chemotactic peptide derived from human monocytes. *Am J Pathol* 1989; **134**: 755–760.
19. Collins PD, Jose PJ, Williams TJ. The sequential generation of neutrophil chemoattractant proteins in acute inflammation in the rabbit *in vivo*. *J Immunol* 1991; **146**: 677–684.
20. Izzo RS, Witkon K, Chen AI, Hadjiyane C, Weinstein MI, Pellechia C. Interleukin-8 and neutrophil markers in colonic mucosa from patients with ulcerative colitis. *Am J Gastroenterol* 1992; **87**: 1447–1452.
21. McElvaney NG, Nakamura H, Birrer P, Hébert CA, Wong WL, Alphonso M, Baker JB, Catalano MA, Crystal RG. Modulation of airway inflammation in cystic fibrosis. *In vivo* suppression of interleukin-8 levels on the respiratory epithelial surface by aerosolization of recombinant secretory leukoprotease inhibitor. *J Clin Invest* 1992; **90**: 1296–1301.
22. Remick DG, DeForge LE, Sullivan JF, Showell HJ. Profile of cytokines in synovial fluid specimens from patients with arthritis. Interleukin-8 and IL-6 correlate with inflammatory arthritides. *Immunol Invest* 1992; **21**: 321–327.
23. Donnely S, Strieter RM, Kunkel SL, Walz A, Robertson CR, Carter DC, Grant IA, Pollok AJ, Haslett C. Interleukin-8 and development of adult respiratory distress syndrome in at risk patients groups. *Lancet* 1993; **341**: 643–647.
24. Miller EJ, Cohen AB, Nagao S, Griffith D, Maunder RJ, Martin TR, Weiner-Kronish JP, Sticherling M, Christophers E, Matthay MA. Elevated levels of NAP-1/interleukin-8 are present in the airspaces of patients with the adult respiratory distress syndrome and are associated with increased mortality. *Am Rev Respir Dis* 1992; **146**: 427–432.
25. Chollet-Martin S, Montravers P, Gibert C, Elbim ?, Desmonts JM, Fagon JY, Gougerot-Pocidalo MA. High levels of interleukin-8 in the blood and alveolar spaces of patients with pneumonia and adult respiratory distress syndrome. *Infect Immun* 1993; **61**: 4556–4559.
26. Hack CE, Mart M, Strack van Schijndel RJM, Eerenberg AJM, Nuijens JH, Thijs LG, Aarden LA. Interleukin-8 in sepsis: relation to shock and inflammatory mediators. *Infect Immun* 1992; **60**: 2835–2842.
27. Friedland JS, Suputtamongkol Y, Remick DG, Chaowagul W, Strieter RM, Kunkel SL, White NJ, Grifin GE. Prolonged elevation of interleukin-8 and interleukin-6 concentrations in plasma and of leukocyte interleukin-8 mRNA levels during septicemic and localized *Pseudomonas pseudomallei* infection. *Infect Immun* 1992; **60**: 2402–2408.
28. Marty C, Misset B, Tamion F, Fitting C, Carlet J, Cavaillon JM. Circulating interleukin-8 concentrations in patients with multiple organ failure of septic and non-septic origin. *Crit Care Med* **22**, 1994; 673–679.
29. Mulligan MS, Jones ML, Bolanowski MA, Baganoff MP, Deppeler CL, Meyers DM, Ryan US, Ward PA. Inhibition of lung inflammatory reactions in rats by an anti-human IL-8 antibody. *J Immunol* 1993; **150**: 5585–5595.
30. Skido N, Mukaida N, Harada ?, Nakanishi I, Watanabe Y, Matsushima K. Prevention of lung reperfusion injury in rabbits by a monoclonal antibody against interleukin-8. *Nature* 1993; **365**: 654–657.
31. Broaddus VC, Boylan AM, Hoeffel JM, Kim KJ, Sadick M, Chuntharapai A, Hébert CA. Neutralization of IL-8 inhibits neutrophils influx in a rabbit model of endotoxin-induced pleurisy. *J Immunol* 1994; **152**: 2960–2967.
32. Chomczynski P, Sacchi N. Single step method of RNA isolation by acid guanidinium thiocyanate phenol chloroform extraction. *Analyt Biochem* 1987; **162**: 156–159.
33. Kasama T, Strieter RM, Lukacs NW, Burdick MD, Kunkel SL. Regulation of neutrophil derived chemokine expression by IL-10. *J Immunol* 1994; **152**: 3559–3569.
34. Cassatella MA, Meda L, Bonora S, Ceska M, Constantin G. Interleukin-10 inhibits the release of pro-inflammatory cytokines from human polymorphonuclear leukocytes. Evidence for an autocrine role of TNF and IL-1β in mediating the production of IL-8 triggered by LPS. *J Exp Med* 1993; **178**: 2207–2211.
35. Wertheim WA, Kunkel SL, Standiford TJ, Burdick MD, Becker FS, Wilke A, Gilbert AR, Strieter RM. Regulation of neutrophil-derived IL-8: the role of prostaglandin E2, dexamethasone and IL-4. *J Immunol* 1993; **151**: 2166–2175.
36. Wang P, Wu P, Anthes JC, Siegel MI, Egan RW, Billah MM. Interleukin-10 inhibits interleukin-8 production in human neutrophils. *Blood* 1994; **83**: 2678–2683.
37. Marie C, Pitton C, Fitting C, Cavaillon JM. IL-10 and IL-4 synergize with TNFα to induce IL-1ra production by human neutrophils. *Cytokine* 1996; **8**: 147–151.
38. Girard D, Paquin R, Naccache PH, Beaulieu AD. Effects of interleukin-13 on human neutrophil functions. *J Leuk Biol* 1996; **59**: 412–419.
39. De Beaux AC, Maingay JP, Ross JA, Fearon KC, Carter DC. Interleukin-4 and interleukin-10 increase endotoxin-stimulated human umbilical vein endothelial cell interleukin-8 release. *J Interferon Cytokine Res* 1995; **15**: 441–445.
40. Samanta AK, Oppenheim JJ, Matsushima K. Interleukin-8 (monocyte-derived neutrophil chemotactic factor) dynamically regulates its own receptor expression on human neutrophils. *J Biol Chem* 1990; **265**: 183–189.
41. Kuhns DB, Gallin JI. Increased cell-associated IL-8 in human exudative and A23187-treated peripheral blood neutrophils. *J Immunol* 1995; **154**: 6556–6562.

ACKNOWLEDGEMENTS. We express our gratitude to J. Oppenheim (NCI) for his gift of the IL-8 cDNA probe. We thank Drs A. Minty and N. Vita (Sanofi Recherche) and J. Abrams (DNAX) for their generous gifts of recombinant human IL-13, anti-IL8 polyclonal antibody and recombinant human IL-10, respectively. We thank D. Fidock for linguistic advice.

**Received 13 June 1996; accepted 17 August 1996**
